# Inferring the phase response curve from observation of a continuously perturbed oscillator

**DOI:** 10.1038/s41598-018-32069-y

**Published:** 2018-09-11

**Authors:** Rok Cestnik, Michael Rosenblum

**Affiliations:** 1 Department of Physics and Astronomy, University of Potsdam, Karl-Liebknecht-Str. 24/25, D-14476 Potsdam-Golm, Germany; 2 Department of Human Movement Sciences, MOVE Research Institute Amsterdam, Vrije Universiteit Amsterdam, van der Boechorststraat 9, Amsterdam, Netherlands; 3 Department of Control Theory, Nizhny Novgorod State University, Gagarin Av. 23, 606950 Nizhny Novgorod, Russia

## Abstract

Phase response curves are important for analysis and modeling of oscillatory dynamics in various applications, particularly in neuroscience. Standard experimental technique for determining them requires isolation of the system and application of a specifically designed input. However, isolation is not always feasible and we are compelled to observe the system in its natural environment under free-running conditions. To that end we propose an approach relying only on passive observations of the system and its input. We illustrate it with simulation results of an oscillator driven by a stochastic force.

## Introduction

Phase response curve (PRC), also known as phase resetting or phase sensitivity curve, is a basic characteristic of a limit cycle oscillator^1–4^. This curve describes variation of the phase *φ* of the system in response to a weak external perturbation *p*(*t*):

1 $$\dot{\phi }=\omega +Z(\phi )p(t).$$

Here *ω* is the natural frequency and *Z*(*φ*) is the oscillator’s PRC. Thus, knowledge of *Z*(*φ*) and *ω* completely determines the phase dynamics for any given weak perturbation. For example, it allows one to determine–at least numerically–the domain of locking to an external force of a given amplitude. Hence, techniques for efficient experimental determination of PRC are in high dema nd.

If it is possible to isolate the oscillator and apply a specially designed perturbation, then determination of PRC is straightforward. To this goal, the experimentalist has to “kick” the system with short and weak pulses at different values of the oscillator’s phase and look for subsequent variation of one or several oscillation periods, i.e., for the evoked asymptotic phase shift^2,4–7^. If the oscillator is noisy–and all real-world systems are–then stimulation for each *φ* shall be performed many times and the results shall be averaged over the trials. Next, the whole procedure shall be repeated for different amplitudes of the stimulation in order to check whether the obtained PRC linearly scales with the amplitude: this would indicate that the chosen stimulation is sufficiently weak (we remind that in theory the PRC is defined for infinitesimally weak perturbations^1,3^).

If one has no control over the applied input and instead has to rely on passive observations, the problem of invoking the PRC is in general not solved and the method will depend on the input. Possibly the best-case scenario is when the input is weak, spiky and with a frequency several times lower than that of the oscillator. Then, as long as the oscillator does not synchronize with the input, the spikes arrive at different phases and never more than one per period. This results in a focused perturbation which provides an estimate of phase shifts for different oscillator phases, like in the standard technique^2,4–7^. If, however, the frequency of the input spike train is higher than that of the oscillator then each period on average gets perturbed more than once, which considerably adds to the complexity of the problem^8^. In general the input may be arbitrary, in particular, it can be a continuous noise-like signal. If both such input and output of the system are measured, then one can infer the PRC using the idea of Spike-Triggered Average (STA)^9^. As has been demonstrated theoretically and numerically for the Hodgkin-Huxley neuronal model, for weak delta-correlated input the STA is proportional to the derivative of the PRC^9^. Recent more practical algorithms exploit weighted STA (WSTA): they imply rescaling of the input within each inter-spike interval to the same length and subsequent averaging with the weights, determined by the length of these intervals^7,10^. Another approach is to use STA with a specific optimal colored noise input^11^.

In this paper we introduce a method for inferring the PRC by fitting the phase model (1) to the observed time series. This nonlinear problem is solved by an iterative procedure that provides frequency *ω* and PRC *Z*(*φ*) as well as instantaneous phase *φ*(*t*). An important novel feature of our algorithm is a built-in error estimation that allows us to monitor the goodness of the reconstruction. We demonstrate the efficiency of the proposed method on several oscillator models driven by correlated noise and compare it with the techniques from refs^7,10^. We show that our technique outperforms them in case of very short time series, not very short correlation and not very weak amplitude of the input.

## Results

### Technique

Suppose we perturb the oscillator under study with stimulation *p*(*t*) and record its output *x*(*t*). For example, *x*(*t*) corresponds to the membrane potential of a cell. Since we assume that the oscillator has a stable limit cycle, without perturbation the process *x*(*t*) would be periodic. In practice, because noise is inevitable, the oscillation would be almost periodic. The technique of extracting the PRC crucially depends on the properties of the signal *x*(*t*) as well as those of the perturbation *p*(*t*). We first discuss the relatively simple case where *x*(*t*) is a smooth signal, suitable for phase estimation with the conventional technique of embedding the signal via the Hilbert transform^12–14^ and performing the protophase-to-phase transformation^15^.

Once an estimate of the phase *φ*(*t*) is obtained, the instantaneous frequency $\dot{\phi }(t)$ can be numerically estimated, and then, representing the PRC as a finite Fourier series,

2 $$Z(\phi )={a}_{0}+\sum _{n=1}^{N}[{a}_{n}\,\cos (n\phi )+{b}_{n}\,\sin (n\phi )],$$

we obtain from Eq. (1) a system of linear equations with respect to the unknown frequency *ω* and the Fourier coefficients *a*_*n*_, *b*_*n*_. Provided we have a good resolution and long enough time series, this system is overdetermined and can be solved by means of optimization, e.g., least squares minimization. Notice that this can be done for an arbitrary form of perturbation *p*(*t*). Thus, a common approach to the problem revolves around estimating the phase *φ* and its derivative $\dot{\phi }$ (the latter requires numerical differentiation and is therefore sensitive to noise). For cases when the signal has a complex form and there is no unique center of rotation in the Hilbert representation, alternative ways of estimating *φ* and $\dot{\phi }$ shall be used. For example, for neural oscillations the phase is often obtained via simple linear interpolation between spikes^7,10,16^. In a different setup, like in studies of the interaction between the respiratory and cardiac oscillators several markers within each cycle of the electrocardiogram are used to determine the phase^17^. Such *ad hoc* techniques proved being useful, albeit they tend to be setup-specific, lack generality, and provide only zero-order approximation of the phase.

Here we introduce a technique that does not require computation of continuous phase *φ* and instantaneous frequency $\dot{\phi }$ from the data. For our approach, in addition to continuous observation of the input forcing *p*(*t*), it is sufficient to define one well-defined event within each cycle of *x*(*t*) such that the times *t*_*m*_ of these events can be considered as the instants of return to a Poincaré surface of section. In the simplest case this event can correspond to a condition *x*(*t*) = const, while generally the problem of choosing a proper Poincaré section is not so trivial and is discussed in more detail below. Correspondingly, these events can be assigned the same value of the phase which without loss of generality can be set to zero, *φ*(*t*_*m*_) = 0. For these events one can choose spikes, threshold crossings, or even bursts (an example can be found in Supplementary material online, Fig. S4). So, in analysis of spiking neurons it is natural to use spikes since they can be reliably detected while the signal between them can be dominated by noise. The phase increase within each inter-event interval, *T*_*m*_ = *t*_*m*+1_ − *t*_*m*_, is defined to be 2*π*.

In what is the *first key step* of our approach, we integrate Eq. (1) over each interval *T*_*m*_, replacing *Z*(*φ*) by its Fourier representation via Eq. (2),

3 $$2\pi =\omega {T}_{m}+{a}_{0}{\int }_{{t}_{m}}^{{t}_{m}+{T}_{m}}p(t){\rm{d}}t+\sum _{n=1}^{N}[{a}_{n}{\int }_{{t}_{m}}^{{t}_{m}+{T}_{m}}p(t)\cos \,[n\phi (t)]{\rm{d}}t+{b}_{n}{\int }_{{t}_{m}}^{{t}_{m}+{T}_{m}}p(t)\sin \,[n\phi (t)]{\rm{d}}t].$$

Certainly, the computation of the right hand side requires knowledge of the phase. Here we point out that in the limit of vanishing perturbations, the phase between events grows nearly linearly. Hence, for a sufficiently weak *p*(*t*) the problem can be quite precisely solved by linearly interpolating the phase *φ*(*t*) = 2*π*(*t* − *t*_*m*_)/*T*_*m*_, inserting it in Eq. (3), numerically computing all the integrals, and solving the linear system by means of optimization, cf.^16^. However, for stronger driving amplitudes the increased inaccuracy of the linear approximation unavoidably translates to inaccuracy of the solution. We address this with the *second key step* in our technique, where the phase is estimated through an iterative procedure, by solving systems (1) and (3) in turns. For the zeroth approximation of the phase we linearly interpolate it between events, *φ*^(0)^(*t*) = 2*π*(*t* − *t*_*m*_)/*T*_*m*_. Then, inserting it in Eq. (3) and solving the system we obtain the first approximation of the solution *ω*^(1)^, *Z*^(1)^. The next approximation of the phase is obtained by numerically integrating Eq. (1), i.e., by solving ${\dot{\phi }}^{(1)}={\omega }^{(1)}+{Z}^{(1)}[{\phi }^{(0)}(t)]p(t)$ separately for each inter-event interval, taking *φ*^(1)^(*t*_*m*_) = 0 for initial conditions. Since the phase *φ*^(0)^, natural frequency *ω*^(1)^, and PRC *Z*^(1)^ are known only approximately, the computed phase at the end of the interval, ${\phi }^{\mathrm{(1)}}({t}_{m}+{T}_{m})={\psi }_{m}^{\mathrm{(1)}}$, generally differs from the correct value 2*π*. Therefore, we *rescale* the phase within each interval: ${\phi }^{\mathrm{(1)}}(t)\to 2\pi {\phi }^{\mathrm{(1)}}(t)/{\psi }_{m}^{\mathrm{(1)}}$. This rescaled phase is then used to obtain the second approximation of the solution *ω*^(2)^, *Z*^(2)^, and then the whole procedure can be repeated to obtain further ones. As illustrated below, the iterations typically converge to the true solution and the quantities ${\psi }_{m}^{(k)}$ can be used to monitor the convergence.

### Numerical tests

First we mention that for clarity of presentation we always set the natural frequency of the oscillator *ω* to 2*π*, thus ensuring the period of an unperturbed oscillator is *T* = 1 (with oscillators where natural frequency is not an explicit parameter this is done by rescaling time). Also, throughout this paper, unless specified differently, we use *k* = 10 iteration steps, *N* = 10 Fourier harmonics, time of simulation *t*_sim_ = 500 and time step d*t* = 0.001.

#### Phase model: proof of concept

We first test the technique on models, where both the true phase and the PRC are known. For this purpose we simulate Eq. (1) with PRCs given as

4 $$Z(\phi )=(1-\,\cos (\phi ))\exp (3[\cos (\phi -\pi /3)-1]),$$

or

5 $$Z(\phi )=-\,\sin (\phi )\exp (3[\cos (\phi -0.9\pi )-\,1]).$$

These curves model PRC of type I and II respectively, as they are classified in the context of neuronal modeling^8,18^. Type I curves are non-negative which means that every stimulus from an excitatory connection shortens the period duration, while type II curves are positive at some phases and negative at others meaning that a stimulus may shorten or lengthen the period depending on when it arrives. We take the input to the system to be an Ornstein-Uhlenbeck process^19^.

6 $$\dot{p}=-\,\frac{p}{\tau }+\varepsilon \sqrt{\mathrm{2/}\tau }\xi (t),$$

where *ξ* is Gaussian white noise $\langle \xi (t)\xi (t^{\prime} )\rangle =\delta (t-t^{\prime} )$, while *ε* and *τ* are the amplitude and correlation time of the driving signal, $\langle p(t)p(t^{\prime} )\rangle ={\varepsilon }^{2}{e}^{-\frac{|t-t^{\prime} |}{\tau }}$. At this point we mention that for the same value of *ε* different oscillators are perturbed by different amounts because they have different PRCs and frequencies. From Eq. (1) we see that the effect of perturbation is proportional to $\varepsilon \parallel Z\parallel /\omega$ where $\parallel \,\cdot \,\parallel$ stands for norm (we use the *L*_2_ functional norm, see Methods). Therefore, to compare the results for different test models and since all frequencies are set to 2*π*, we specify driving strength with the quantity $\varepsilon \parallel Z\parallel$.

Spike data is generated using the condition *φ*(*t*_*m*_) = *m* ⋅ 2*π*. Then, we perform *k* iterations of the reconstruction procedure and compare the results against the data. To quantify the quality of the reconstruction we compute the difference between the recovered and true PRCs, Δ_*Z*_, and average deviation of *ψ*_*m*_ from 2*π*, Δ_*ψ*_ (see Eqs (10) and (12) in Methods). Δ_*Z*_ should be compared to 1, where ${\Delta }_{Z}\ll 1$ means the reconstructed PRC matches the true one closely, while Δ_*Z*_ ≈ 1 signals that the reconstructed PRC does not resemble the true one at all. Δ_*ψ*_ is to be compared to a related measure of event irregularity, ${{\rm{\Delta }}}_{{\psi }_{T}}$ (see Eq. (13) in Methods), where ${\Delta }_{\psi }\ll {{\rm{\Delta }}}_{{\psi }_{T}}$ means that our reconstruction can reproduce the data well, while ${{\rm{\Delta }}}_{\psi }\approx {{\rm{\Delta }}}_{{\psi }_{T}}$ signals that, statistically speaking, the reconstructed PRC and frequency are not able to reproduce the data any more accurately than a perfectly periodic oscillator with frequency $\langle \omega \rangle =\langle \frac{2\pi }{{T}_{m}}\rangle$.

**Figure 1:**
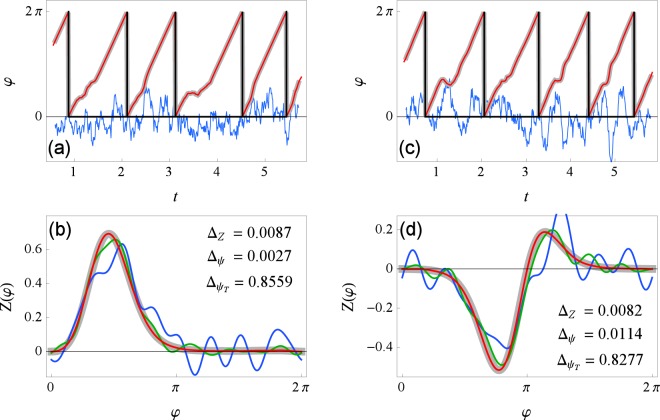
A reconstruction of phase *φ* and PRC *Z* for PRC type I (**a**,**b**) and type II (**c**,**d**). In the top plots (**a**,**c**), true phase is plotted in thick gray, reconstructed phase in red, driving signal in blue (scaled for being visually comparable to the phase) and times of spikes *t*_*m*_ in black. In the bottom plots (**b**,**d**), true PRC is plotted in thick gray, first iteration *Z*^(1)^ in blue, second *Z*^(2)^ in green and tenth *Z*^(10)^ in red. Parameters are $\varepsilon \parallel Z\parallel =5$ and *τ* = 0.1.

**Figure 2:**
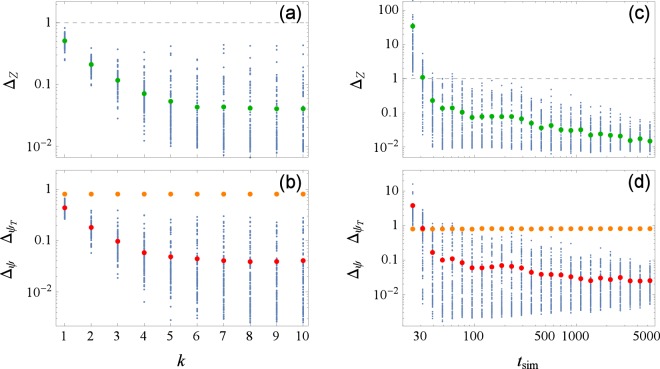
Errors of reconstruction Δ_*Z*_ and Δ_*ψ*_, Eqs (10) and (12), versus iteration number *k* (**a**,**b**) and versus the duration of data recordings *t*_sim_ (**c**,**d**), for PRC type II. For each *k* and each *t*_sim_, 100 points corresponding to different realizations of noise are plotted with blue dots and their average in colors: Δ_*Z*_ in green, Δ_*ψ*_ in red (and ${{\rm{\Delta }}}_{{\psi }_{T}}$ in orange for reference). Parameters are $\varepsilon \parallel Z\parallel =5$, *τ* = 0.1, *t*_sim_ = 500 (**a**,**b**) and *k* = 10 (**c**,**d**).

In Fig. 1 we show a reconstruction example for the first test. We use fairly strong driving $\varepsilon \parallel Z\parallel =5$ and moderate correlation *τ* = 0.1 so that the phase alterations are visible in the plot. Notice that the error measures indicate a good reconstruction, i.e., ${\rm{\Delta }}Z\ll 1$ and ${{\rm{\Delta }}}_{\psi }\ll {{\rm{\Delta }}}_{{\psi }_{T}}$. Notice also that the initial PRC estimate *Z*^(1)^ is quite imprecise while further iterations improve in precision and converge to the true curve. To see this convergence in more detail we plot the error measures for each iteration *k* in Fig. 2(a,b), for PRC type II. We also investigated how the error depends on the duration of data recordings *t*_sim_ and found that a few hundred periods suffice for a reliable reconstruction, see Fig. 2(c,d).

#### The Morris-Lecar neuron: effect of amplitude *ε* and correlation time *τ*

Our next test model is the Morris-Lecar neuronal oscillator^3,20^

7 $$\begin{array}{rcl}\dot{V} & = & I-{g}_{L}(V-{V}_{L})-\,{g}_{K}\,w(V-{V}_{K})-\,{g}_{Ca}\,{m}_{\infty }(V)(V-{V}_{Ca})+p(t),\\ \dot{w} & = & \lambda (V)({w}_{\infty }(V)-w),\end{array}$$

where

8 $$\begin{array}{rcl}{m}_{\infty }(V) & = & [1+\,\tanh ((V-{V}_{1})/{V}_{2})]/2,\\ {w}_{\infty }(V) & = & [1+\,\tanh ((V-{V}_{3})/{V}_{4})]\mathrm{/2},\\ \lambda (V) & = & \cosh \,[(V-{V}_{3})/(2{V}_{4})]/3,\end{array}$$

and the parameters are: *I* = 0.07, *g*_*L*_ = 0.5, *g*_*K*_ = 2, *g*_*Ca*_ = 1.33, *V*_1_ = −0.01, *V*_2_ = 0.15, *V*_3_ = 0.1, *V*_4_ = 0.145, *V*_*L*_ = −0.5, *V*_*K*_ = −0.7 and *V*_*Ca*_ = 1. A reconstruction depiction similar to Fig. 1 can be found in Supplementary material online, Fig. S9.

**Figure 3:**
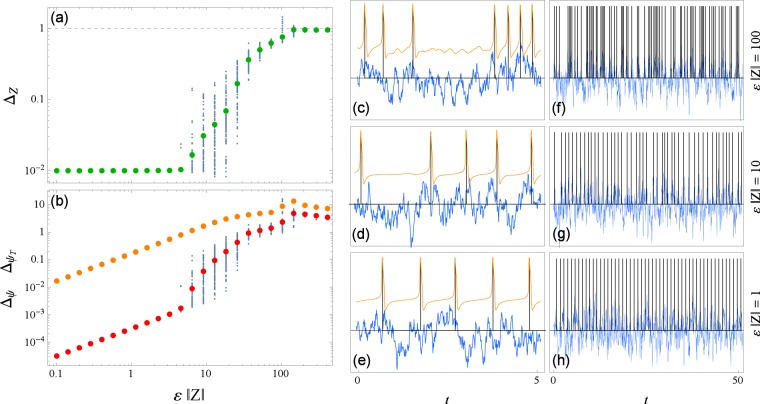
The dependence of errors of reconstruction Δ_*Z*_ and Δ_*ψ*_, Eqs (10) and (12), on driving amplitude *ε* for the Morris-Lecar neuronal oscillator Eq. (7). In (**a**,**b**) for each value of *ε*, 100 points corresponding to different realizations of noise are plotted with blue dots and their average in colors: Δ_*Z*_ in green, Δ_*ψ*_ in red (and ${{\rm{\Delta }}}_{{\psi }_{T}}$ in orange for reference). Shown in (**c**–**h**) are segments of signal on two time scales for three amplitudes of driving $\varepsilon \parallel Z\parallel$. In orange the voltage *V*(*t*) (only in (**c**–**e**)) and in blue the input signal *p*(*t*), scaled for being visually comparable. The times of zero phase *t*_*m*_ are marked with vertical black lines. The correlation time is *τ* = 0.1.

We investigate how amplitude of driving *ε* effects the reconstruction. For that purpose we simulate a forced oscillator with different values of *ε* and compute the errors of reconstruction Δ_*Z*_, Eq. (10), and Δ_*ψ*_, Eq. (12), see Fig. 3. The true PRC of the Morris-Lecar system is obtained via the standard technique, see Methods and ref.^7^. For weak driving the PRC error Δ_*Z*_ is small and independent of amplitude, while Δ_*ψ*_ and ${{\rm{\Delta }}}_{{\psi }_{T}}$ scale approximately linearly with amplitude and maintain a constant ratio, ${{\rm{\Delta }}}_{\psi }/{{\rm{\Delta }}}_{{\psi }_{T}}\approx {\rm{const}}\ll 1$. The reconstruction works well, as expected. As the driving amplitude increases, at some point Δ_*Z*_ begins to grow and Δ_*ψ*_ starts to approach ${{\rm{\Delta }}}_{{\psi }_{T}}$, a sign of declining reconstruction quality (in Fig. 3 this happens around $\varepsilon \parallel Z\parallel \approx 5$). In this regime the distribution of errors (both Δ_*Z*_ and Δ_*ψ*_) widens, meaning that reconstruction quality for the same parameters may vary considerably from trial to trial. As the driving gets very strong (around $\varepsilon \parallel Z\parallel \approx 100$ in Fig. 3) the reconstruction does not work, i.e., Δ_*Z*_ ≈ 1 and ${{\rm{\Delta }}}_{\psi }\approx {{\rm{\Delta }}}_{{\psi }_{T}}$. As a side note, in Fig. 3(b) one can observe what looks like a change in behavior around $\varepsilon \parallel Z\parallel \approx 100$, where there is a jump in Δ_*ψ*_ and ${{\rm{\Delta }}}_{{\psi }_{T}}$. This is due to the test model we use, Morris-Lecar, and its behavior when strongly stimulated (at that amplitude the system spends some time in the vicinity of the fixed point) and is not a consequence of our technique (in Supplementary material online one can see analogous plots for other oscillators where this is not observed, Fig. S9).

**Figure 4:**
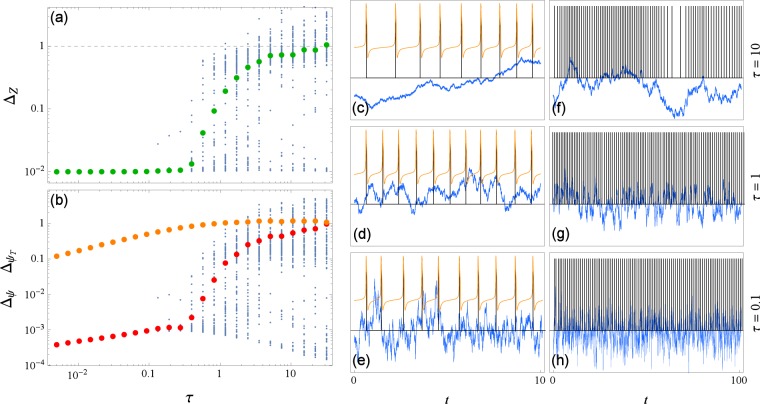
The dependence of errors of reconstruction Δ_*Z*_ and Δ_*ψ*_, Eqs (10) and (12), on correlation time *τ* of the driving for the Morris-Lecar oscillator, Eq. (7). In (**a**,**b**) for each value of *τ*, 100 points corresponding to different realizations of noise are plotted with blue dots and their average in colors: Δ_*Z*_ in green, Δ_*ψ*_ in red (and ${{\rm{\Delta }}}_{{\psi }_{T}}$ in orange for reference). Shown in (**c**–**h**) are segments of signal on two time scales for three values of correlation time *τ*. In orange the voltage *V*(*t*) (only in (**c**–**e**)) and in blue the input signal *p*(*t*), scaled for being visually comparable. The times of zero phase *t*_*m*_ are marked with vertical black lines. The driving strength is $\varepsilon \parallel Z\parallel =3$.

In the same way as for the amplitude, we now investigate how correlation time *τ* effects the reconstruction, see Fig. 4. The reconstruction works best for short correlation times: Δ_*Z*_ as well as the ratio ${{\rm{\Delta }}}_{\psi }/{{\rm{\Delta }}}_{{\psi }_{T}}$ are small and roughly constant, but in this case Δ_*ψ*_ and ${{\rm{\Delta }}}_{{\psi }_{T}}$ grow sublinearly with *ε*. Similarly to what we have seen in the previous figure, as we increase *τ* there comes a point where Δ_*Z*_ starts to grow and Δ_*ψ*_ starts to approach ${{\rm{\Delta }}}_{{\psi }_{T}}$ (this happens around *τ* ≈ 0.4 in Fig. 4). Likewise the distribution from this point on widens, indicating that reconstruction quality varies from trial to trial. As we continue to increase *τ* there comes a point at which average Δ_*Z*_ reaches 1 and Δ_*ψ*_ reaches ${{\rm{\Delta }}}_{{\psi }_{T}}$ (this happens around *τ* ≈ 30 in Fig. 4). Interestingly, we can see that while the error averages in general reflect a bad reconstruction, there persists a branch of reconstructions that maintains a low error which is even slightly decreasing with *τ*. This means that with a small probability we can still get a good reconstruction from slow varying input (an example can be found in Supplementary material online, Fig. S3), and by monitoring the solution errors we can distinguish successful reconstructions from the unsuccessful ones. A similar qualitative description is valid for other oscillators in this context as well (analogous analysis on other oscillators can be found in Supplementary material online, Fig. S9). Here it is worth mentioning that while our chosen input, Eq. (6), yields white noise with zero intensity in the limit *τ* → 0, we have tested our procedure for white noise with finite intensity and it works as well (an example can be found in Supplementary material online, Fig. S1).

Up to this moment we have been postponing a discussion of a crucial problem, namely how to determine the instants *t*_*m*_ corresponding to zero phase, *φ*(*t*_*m*_) = 0. For spiky data where only the times of events can be reliably detected (so that the data is represented by a point process) there is no other option but to use the times of events. This corresponds to pure phase models, where this problem does not exist and the values *t*_*m*_ are obtained in the course of simulation. This is not the case for full models, where both phases and amplitudes shall be taken into account. So, for the analysis of the Morris-Lecar model we used a simple threshold rule and defined zero phase from the condition *x*(*t*_*m*_) = *x*_min_ + 0.9(*x*_max_ − *x*_min_), $\dot{x}({t}_{m}) < 0$, where *x*_min_ and *x*_max_ are the minimal and maximal measured value of the observed signal. However, this is not exact: in fact, the proper section of the limit cycle shall coincide with a line (or, generally, surface) of constant phase, called isochrone^21^, but in experimental conditions the model equations and isochrones are not known *a priori*. Nevertheless, for neuronal oscillators this is not a complication if the threshold is chosen close to the maximum of the pulse, i.e., in the domain of fast motion along the limit cycle. Here the density of isochrones is low, i.e., phase gradient in the direction perpendicular to the isochrones is small (for an illustration of the isochrone structure see Supplementary material online, Fig. S6b). Respectively, the error due to deviation of the line *x* = const from the isochrone is small as well. For general oscillators this is not true, and the problem requires a special treatment, illustrated by the following example.

#### The van der Pol oscillator: importance of choosing a proper Poincaré section

Our third test system is the van der Pol oscillator^22^

9 $$\ddot{x}-2(1-{x}^{2})\dot{x}+x=p(t).$$

With this model we address the importance of choosing an appropriate Poincaré section for determination of the beginnings of each cycle, i.e., instants *t*_*m*_ corresponding to zero phase *φ*(*t*_*m*_) = 0. Here we make use of the ability of our technique to monitor the error of reconstruction.

**Figure 5:**
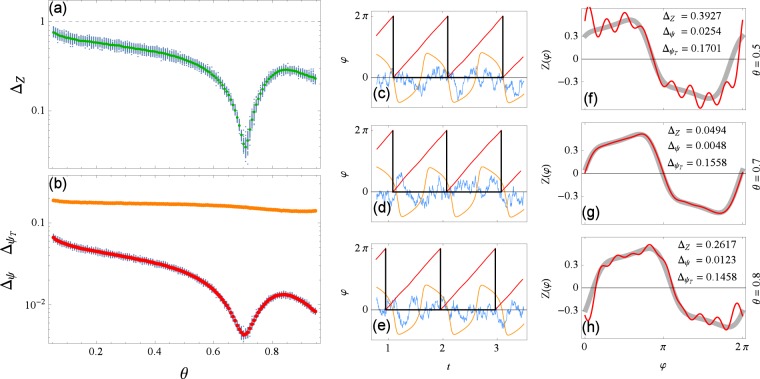
The dependence of errors of reconstruction Δ_*Z*_ and Δ_*ψ*_, Eqs (10) and (12), on the chosen Poincaré section, determined by the threshold parameter *θ* (see Methods), for the van der Pol oscillator, Eq. (9). In (**a**,**b**) for each value of *θ*, 100 points corresponding to different realizations of noise are plotted with blue dots and their average in colors: Δ_*Z*_ in green, Δ_*ψ*_ in red (and ${{\rm{\Delta }}}_{{\psi }_{T}}$ in orange for reference). Shown in (**c**–**e**) are segments of signal for three values of *θ*. Variable *x*(*t*) in orange and the input *p*(*t*) in blue, scaled for being visually comparable. The times of zero phase *t*_*m*_ are marked with vertical black lines and in red is the reconstructed phase. In (**f**–**h**) the corresponding PRC reconstructions are plotted with red. The true PRC is depicted with a thick gray curve. Parameters are $\varepsilon \parallel Z\parallel =1$ and *τ* = 0.1.

**Figure 6:**
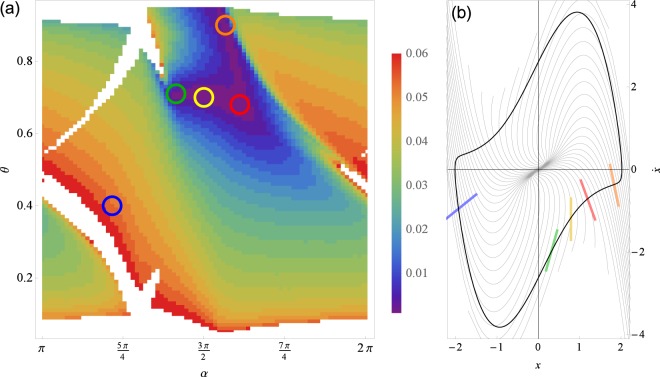
The error of reconstruction Δ_*ψ*_, Eq. (12), with respect to the inclination of the Poincaré surface of section *α*, and its relative position *θ*, see Methods for definition. In (**a**) the white regions correspond to error values out of range ${{\rm{\Delta }}}_{\psi } > 0.06$. The particular values marked with colored circles correspond to example surfaces of section plotted in (**b**) with lines of the same color. The red (Δ_*ψ*_ = 0.0045), green (Δ_*ψ*_ = 0.0051), yellow (Δ_*ψ*_ = 0.0049) and orange (Δ_*ψ*_ = 0.0051) sections yield good period determination: the error is low and in (**b**) we see that they have a similar inclination to the local isochrones, plotted with thin gray curves. The blue one (Δ_*ψ*_ = 0.054) is an example of an inaccurate surface of section that does not correspond to local isochrones (see (**b**)) and therefore yields a high error. Notice that the yellow line corresponds to the optimal section in Fig. 5. The search was performed using a single simulation run of length *t*_sim_ = 500 (corresponding to roughly 500 periods). Parameters are $\varepsilon \parallel Z\parallel =1$ and *τ* = 0.1.

As a first step we probe different threshold values for the signal *x*(*t*) and choose the one which yields the smallest error Δ_*ψ*_. For this purpose we parametrize the threshold values with a parameter $0 < \theta  < 1$, see Methods for details, and perform a search over *θ*. For each value of the threshold *θ*, 100 simulations with different noise realizations were performed. We have calculated both the error of the PRC and the Δ_*ψ*_ error. We underline, that Δ_*ψ*_ can be calculated from data alone, without any *a priori* knowledge of the system. The results are shown in Fig. 5. A clear minimum in both errors around the value *θ* = 0.7 indicates the optimal threshold that corresponds to the surface of section tangential to the local isochrone, see Fig. 6b.

Generally, the goodness of the surface of section (a line in this example) is determined by two factors: the line should be tangential to an isochrone and it should be chosen in the domain of fast motion. This cannot always be ensured by simple thresholding, *x*(*t*_*m*_) = const. Therefore, we consider inclined lines of section, corresponding to local linear approximation of isochrones. This can be done even if we have access to only one variable, in our case *x*(*t*). As is well-known, a phase portrait, topologically equivalent to the true one, can be obtained from a single time series via computation of the derivative or time-delayed embedding^23,24^. We use here the former option, computing $\dot{x}(t)$ using the five point finite difference scheme^25^. In the two-dimensional embedding with coordinates $(x,\dot{x})$ we then search for an optimal linear section, determined by its position *θ* and inclination *α*, see Methods for details. We use a single simulated time series with the total length of 500 periods, mimicking a real world scenario with limited collected data available, and compute the error Δ_*ψ*_, for each pair of values *θ*, *α*. In this way a possibly better surface of section is found, see Fig. 6. This approach can be further improved by a high-dimensional search, e.g., by locally approximating isochrones with a higher order polynomial.

#### Comparison with the WSTA technique

**Figure 7:**
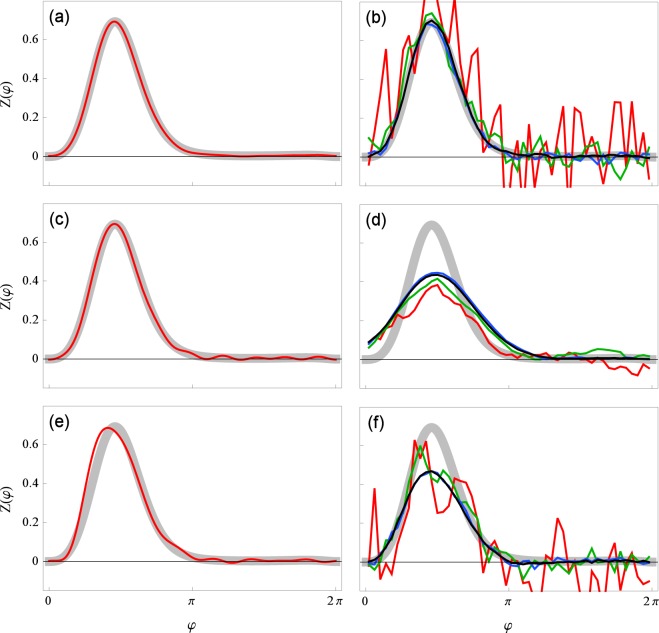
Comparison of our method (**a**,**c**,**e**) and the WSTA method^7,10^ (**b**,**d**,**f**). The data was simulated using the phase model (1). Different colors correspond to the length of time series used for the reconstruction: *t*_sim_ = 10^2^ in red, 10^3^ in green, 10^4^ in blue and 10^5^ in black (for our method (**a**,**c**,**e**) only the *t*_sim_ = 10^2^ is plotted). The true PRC is depicted with a thick gray curve. In (**a**,**b**) the coupling is relatively weak, $\varepsilon \parallel Z\parallel =5$, and the correlation time of the input is relatively small, *τ* = 0.01. These are good conditions for both methods, and indeed both perform well. In (**c**,**d**) the coupling remains as before but the correlation time is larger, *τ* = 0.1. In (**e**,**f**) the coupling strength is increased, $\varepsilon \parallel Z\parallel =20$, while the correlation time is small again, *τ* = 0.01.

In the last test we compare the performance of out approach with that of the WSTA method^7,10^, see Fig. 7. As expected, our technique performs better if the correlation time and/or amplitude of the input is relatively large. This is due to the fact that, in contradistinction to WSTA, we do not use the assumptions of delta-correlated input and of linearly growing phase. Moreover, the test demonstrates that our technique works better for shorter time series.

## Discussion

To summarize, in this paper we introduce a new method for obtaining the PRC and natural frequency of an oscillator relying only on observation of the oscillator’s signal and its continuous perturbation. We demonstrate its efficiency by recovering the PRC of several model systems driven by correlated noise, from only a few hundred observed periods. Furthermore, we provide error measures indicating the quality of the inference that can be calculated from data, without any additional knowledge of the system.

We then explore the effects of the amplitude and correlation time of the driving signal. Generally, the reconstruction works best for perturbation with low amplitude and short correlation time, although the requirements here are not so crucial as in the case of the WSTA technique^7,10^. The degradation of the model inference at higher amplitudes can be due to two reasons: the phase description of an oscillator in terms of Eq. (1) loses its validity or/and linear approximation of the phase dynamics becomes too poor for the iterative scheme to converge. The latter problem may be solved by using a different initial estimate of the phase, e.g., obtained via the Hilbert transform. On the other hand, the degradation of the reconstruction procedure at large correlation times occurs due to a limitation of our technique. Indeed, if the driving is very slow and can be approximately considered as constant within one oscillation period, then the integrals in Eq. (3) vanish and the whole procedure fails. However, for some realizations of noise, relatively strong and slow driving yields a good reconstruction as well (see examples in Supplementary material online, Figs S2 and S3) and the introduced error measures allow us to examine whether the reconstruction is good or not. Finally, we mention that in the white noise limit an additional term, proportional to the square of noise intensity, appears in Eq. (1)^26^. This fact can be another source of error for large perturbation amplitudes.

Finally, we analyze the importance of proper determination of the points that can be considered as beginning of the periods, i.e., the points that are assigned zero phase. This tends to be straightforward for most neuronal models where spikes are the natural choice both due to their detection being robust to noise and the fact that during a spike the phase gradient is typically small dismissing the importance of the shape of the chosen surface of section. In general though, the choice of an appropriate surface of section is crucial. To this end we propose to search for an optimal Poincaré section which minimizes the reconstruction error Δ_*ψ*_. For this search we parameterize these sections by two quantities, namely their position and inclination, use a two-dimensional embedding of the measured time series, and vary these quantities to find the combination that yields minimal Δ_*ψ*_. In this way we find local approximations of the isochrones by straight lines (cf Fig. S8 in Supplementary material online). This approach can be further extended to a nonlinear fit of isochrones. For high-dimensional systems the performance of the PRC estimation can be further improved by using a three-dimensional embedding and approximating the isochrones by inclined planes, etc. Alternatively, for a long data set one can reveal the isochrone structure by estimating the surfaces with the mean first return time equal to the period^27^. Next, our approach can be also combined with the technique suggested in the framework of WSTA in ref.^7^. There the authors addressed the problem of choosing the proper section by rescaling and averaging *n* > 1 consecutive periods instead of one. The inaccuracy due to deviation of the used Poincaré section from the true isochrone is then distributed over *n* periods. For our approach the inter-event intervals *T*_*m*_ = *t*_*m*+1_ − *t*_*m*_ shall be simply replaced by ${T}_{m}^{(n)}={t}_{m+n}-{t}_{m}$, and the left hand side of Eq. (3) shall be changed to *n* ⋅ 2*π*. However, other numerical errors accumulate, and therefore we do not expect this approach to be superior than the search for an optimal section.

## Methods

### Reconstruction errors

For quantification of how well our reconstructed PRC resembles the true one we use the following expression

10 $${{\rm{\Delta }}}_{Z}=(\parallel {Z}^{(t)}-{Z}^{(r)}\parallel )/\parallel {Z}^{(t)}\parallel ,$$

where *Z*^(t)^ refers to the true PRC, *Z*^(r)^ to the reconstructed one and $\parallel \,\cdot \,\parallel$ is the *L*_2_ function norm: $\parallel f\parallel ={[{\int }_{0}^{2\pi }{f}^{2}(\phi ){\rm{d}}\phi ]}^{\mathrm{1/2}}$. When the reconstructed PRC nearly coincides with the true one the error is small, i.e., ${{\rm{\Delta }}}_{Z}\ll 1$. When the two PRCs are not similar, but are of the same order of magnitude, Δ_*Z*_ is of the order of 1, but in general Δ_*Z*_ can have arbitrarily large values. For computation of the true PRC we first determine the period of the autonomous limit cycle oscillation, *T*_0_, using the Henon trick^28^. Next, similarly to^7^, we instantaneously perturb the oscillator at different phases within one period (phases are taken proportionally to the time from the beginning of the cycle) and look for the shift in the asymptotic phase. If the period where perturbation arrives is denoted by *T*_1_ and the following periods as *T*_2_, *T*_3_, …, then the phase shift normalized to the perturbation strength *ε* is

11 $${Z}^{(t)}(\phi )=2\pi \frac{n{T}_{0}-\sum _{i=1}^{n}{T}_{i}}{\varepsilon {T}_{0}}.$$

The number of periods *n* required for a complete relaxation to the limit cycle can be easily chosen by trial and error by checking that the result does not depend on *n*.

Next, we quantify how well our reconstruction describes the system which generated the data. *ψ*_*m*_ is defined as the reconstructed phase at the end of interval *m* which in general differs from the true phase of 2*π* due to the inaccuracy of the reconstructed PRC and *ω*. Therefore the average deviation of *ψ*_*m*_ from 2*π* is a natural measure of goodness for our reconstruction

12 $${{\rm{\Delta }}}_{\psi }={\langle {({\psi }_{m}-2\pi )}^{2}\rangle }^{\mathrm{1/2}},$$

where $\langle \cdot \rangle$ refers to averaging over *m*. It can be computed solely from data (unlike Δ_*Z*_ which requires the knowledge of the true PRC) meaning that it can be used in real experiments. However, Δ_*ψ*_ should not be taken as an absolute measure because it also depends on the inherent irregularity of inter-event intervals. With that in mind we define a measure of event irregularity

13 $${{\rm{\Delta }}}_{{\psi }_{T}}={\langle {(\langle \omega \rangle {T}_{m}-2\pi )}^{2}\rangle }^{\mathrm{1/2}},$$

where $\langle \omega \rangle =\langle \frac{2\pi }{{T}_{m}}\rangle$ is the mean frequency. This measure serves as a good benchmark since it can be understood as Δ_*ψ*_ calculated for a trivial reconstruction, namely, when average period is used as a prediction of next inter-event interval. Hence, an indication of good reconstruction is the condition ${{\rm{\Delta }}}_{\psi }\ll {{\rm{\Delta }}}_{{\psi }_{T}}$.

### A choice of Poincaré section

In the simplest case the same phase is assigned to the instants when the signal crosses a chosen threshold from above (the crossings from bellow can be taken as well so long as we are consistent). Practically, this instants are determined by linear interpolation between the closest values above and below the threshold. In this work we parameterize a threshold value *s*_thr_ by *θ* ∈ (0, 1), so that *s*_thr_ = *s*_min_ + *θ*(*s*_max_ − *s*_min_), where *s*_min_ and *s*_max_ are the minimum and maximum of the considered signal. With the embedded signal, *x*(*t*), $\hat{x}(t)$, we choose a Poincaré section as a straight line at an angle *α* (in our case we use $\hat{x}=\dot{x}$). This is accomplished by rotating the embedded limit cycle by the angle *α*, such that the line of section becomes parallel to the horizontal axis and then taking the vertical axis projection of the limit cycle as an auxiliary signal: ${s}_{{\rm{aux}}}=-\,x(t)\sin (\alpha )+\hat{x}(t)\cos (\alpha )$. Then the auxiliary signal is thresholded as before. A depiction of the process may be found in Supplementary material online, Fig. S7.

## Electronic supplementary material

Supplementary material
