# Supplementary material for: Inferring the phase response curve from observation of a continuously perturbed oscillator
Source: Sci Rep. 2018 Sep 11;8:13606. doi: 10.1038/s41598-018-32069-y (PMC6134126; doi:10.1038/s41598-018-32069-y)
Supplement: Supplementary file 1 — Supplementary material [file 41598_2018_32069_MOESM1_ESM.pdf]

Inferring the phase response curve from observation of a continuously perturbed oscillator - Supplementary material

Rok Cestnik[1,2] **and Michael Rosenblum**[1,3,*]

[1]Department of Physics and Astronomy, University of Potsdam, Karl-Liebknecht-Str. 24/25, D-14476 Potsdam-Golm, Germany
[2]Department of Human Movement Sciences, MOVE Research Institute Amsterdam, Vrije Universiteit Amsterdam, van der Boechorststraat 9, Amsterdam, Netherlands
[3]Department of Control Theory, Nizhny Novgorod State University, Gagarin Av. 23, 606950, Nizhny Novgorod, Russia

White noise driving

Here we demonstrate a reconstruction with Gaussian white noise input $\langle p(t)p(t')\rangle = \varepsilon^2\delta(t-t')$, see Fig. S1. We simulate the system by integrating Eq. (1) in the main text with the Milstein scheme[1] using the PRCs introduced in the main text, Eqs. (4) and (5). We record the differences of the Wiener process ΔW as the driving signal.

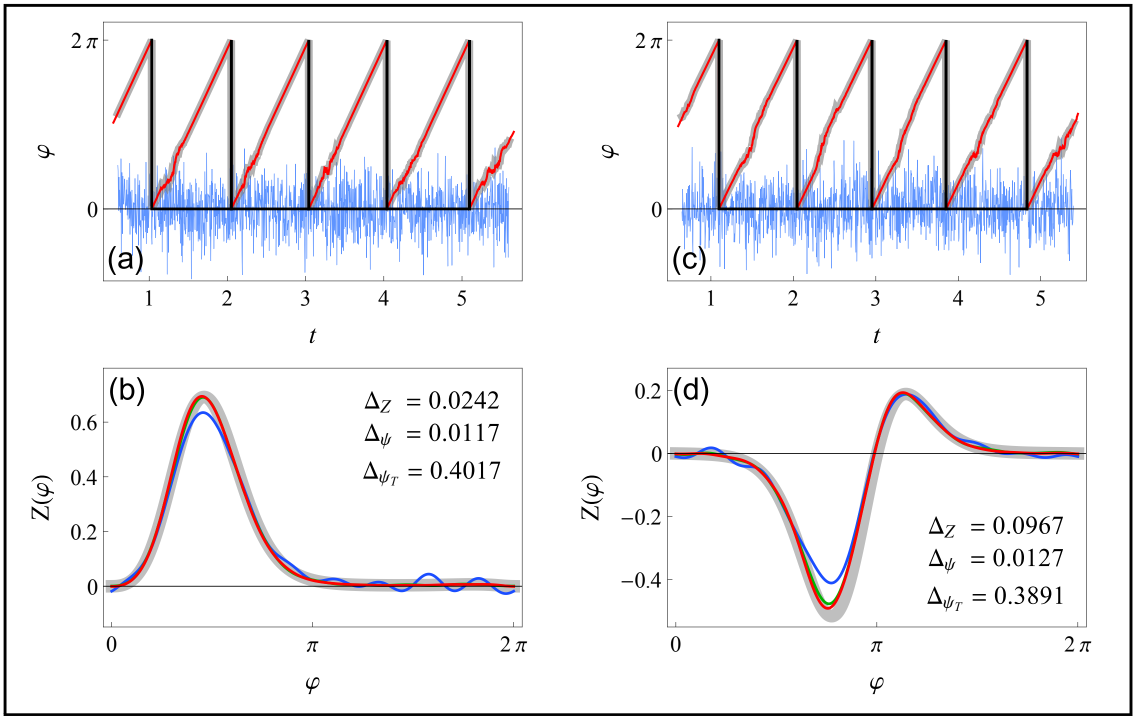

Figure S1. A reconstruction for a phase oscillator driven by white noise (analogous to Fig. 1 in the main text, (a, c) in blue differences of the Wiener process ΔW instead of signal p). The driving strength is $\varepsilon\|Z\| = 1$.

The Morris-Lecar neuron: examples of strong: $\varepsilon\|Z\| = 20$, and slow: $\tau = 10$ driving

On average, cases with strong and slow driving yield bad reconstructions, however, this can vary from case to case. Here we show examples of good reconstructions for those circumstances, see Fig. S2 for strong and Fig. S3 for slow driving. They were chosen as the best from 20 trials (in terms of error measures, Eqs. (10), (12) and (13) introduced in the main text).

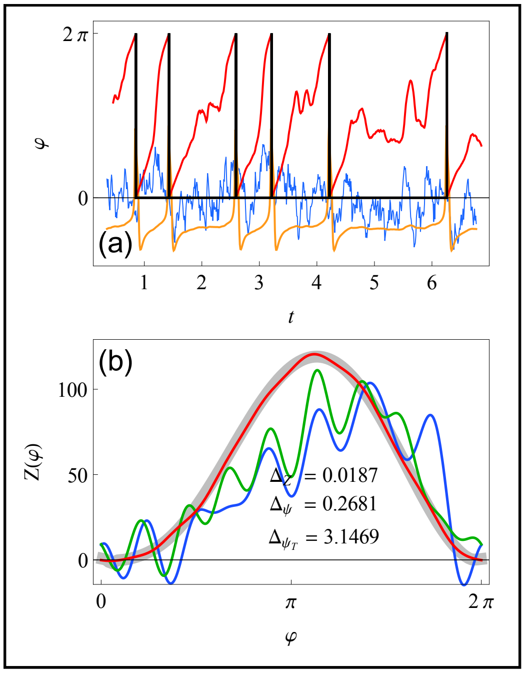

Figure S2. An example of a reconstruction with strong driving, Morris-Lecar neuron (similar to Fig. 1 in the main text). Parameters are $\varepsilon\|Z\| = 20$ and $\tau = 0.1$.

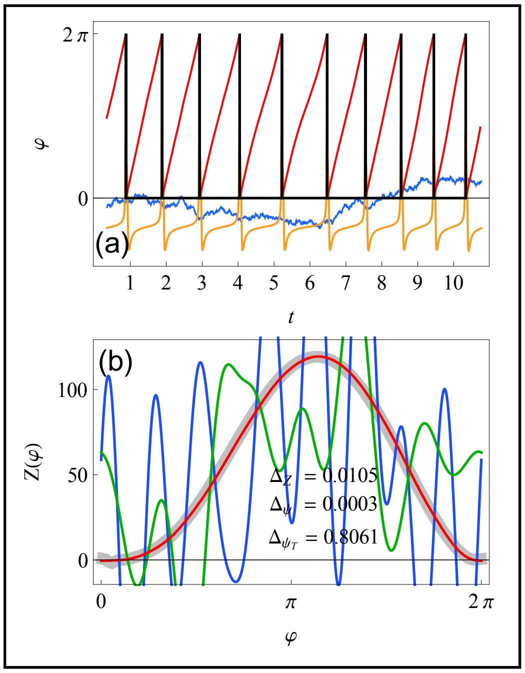

Figure S3. An example of a reconstruction with slow driving, Morris-Lecar neuron (similar to Fig. 1 in the main text). Parameters are $\varepsilon\|Z\| = 3$ and $\tau = 10$.

The Hindmarsh-Rose neuron: example on bursting

To further support the argument that the data our method requires is general we perform a reconstruction on a bursting neuron. We use the Hindmarsh-Rose neuronal model[2]:

$$\begin{aligned}\dot{x} &= I + y - ax^3 + bx^2 - z + p(t)\ ,\\ \dot{y} &= c - dx^2 - y\ ,\\ \dot{z} &= r[s(x - x_R) - z]\ ,\end{aligned} \tag{1}$$

the parameters are: $I = 1.28$, $a = 1$, $b = 3$, $c = 1$, $d = 5$, $r = 0.0006$, $s = 4$ and $x_R = -1.6$. In this regime the neuron is bursting periodically. For the reconstruction we use parameters $\varepsilon\|Z\| = 0.01$, $\tau = 0.01$, $t_{\text{sim}} = 1000$, $\mathrm{d}t = 0.0001$ and $N = 15$. The beginning of the period is determined by the time of the first spike in a burst (this is arbitrary, any of the spikes could be considered for this role). See Fig. S4 for a reconstruction depiction. The PRC of this bursting cycle has a very sharp feature around the phase $\varphi \approx 1$ where the bursting stops. This is hard to capture with our method since we take the Fourier representation of the PRC and steep features require several harmonics to be expressed. Taking many harmonics can be impractical since each harmonic yields an unknown constant needed to be optimized, which can make the data requirements considerably big. With $N = 15$ Fourier harmonics that feature is to some degree smeared in phase, but still apparent.

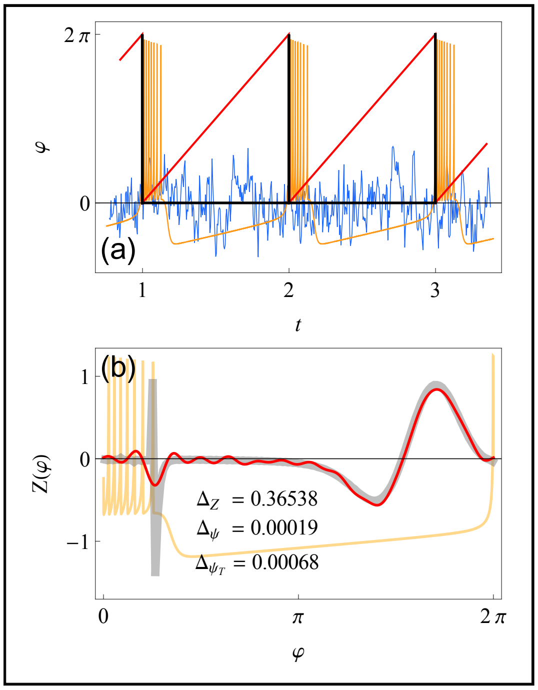

Figure S4. An example of a reconstruction using bursts as the events with equal phase, Hindmarsh-Rose neuron (similar to Fig. 1 in the main text). Parameters are $\varepsilon\|Z\| = 0.01$ and $\tau = 0.01$.

The van der Pol oscillator: varying the number of Fourier harmonics N

We explore the effect of using different numbers of Fourier harmonics N. The PRC of the oscillator for the chosen parameters can not be captured well by a single harmonic term but we also know that using more terms means more unknowns to fit and therefore a necessity for more data to constrain them. By increasing the number of Fourier harmonics N while keeping the length of the time series t_{sim} constant we therefore expect the error to have a minimum, see Fig. S5.

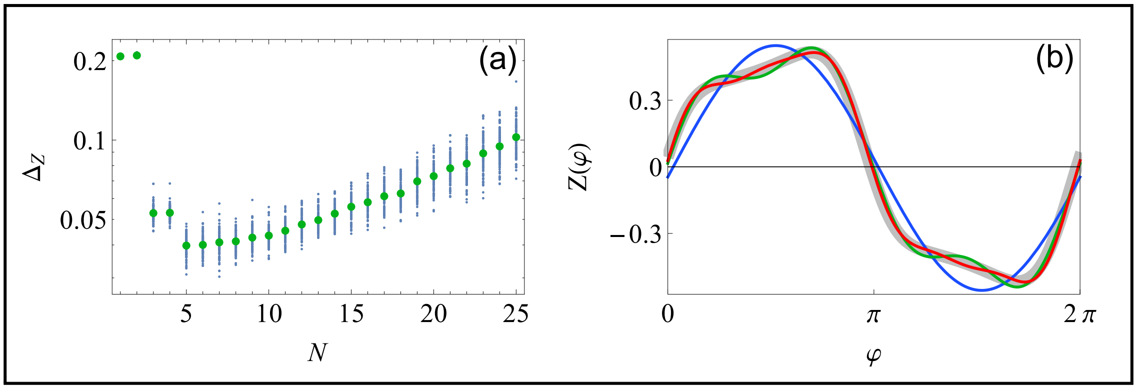

Figure S5. The dependence of PRC error Δ_Z, Eq. (10) in the main text, on the number of Fourier harmonics N for the van der Pol oscillator Eq. (9) in the main text. In (a) for each value of N, 100 points corresponding to different realizations of noise are plotted with blue dots and their average in green. In (b) PRCs reconstructed using $N = 1$, 3 and 5 in blue, green and red respectively (true PRC in thick gray). Parameters are $\varepsilon\|Z\| = 1$ and $\tau = 0.1$.

Choice of a proper Poncaré section

In the case of a forced oscillator, the correct Poincaré section for determining instants of same phase corresponds to an isochrones surface. Here we show the isochrone structure of the two oscillators used in the main text, van der Pol and Morris-Lecar, see Fig. S6. The isochrones were computed using the backward integration method[3].

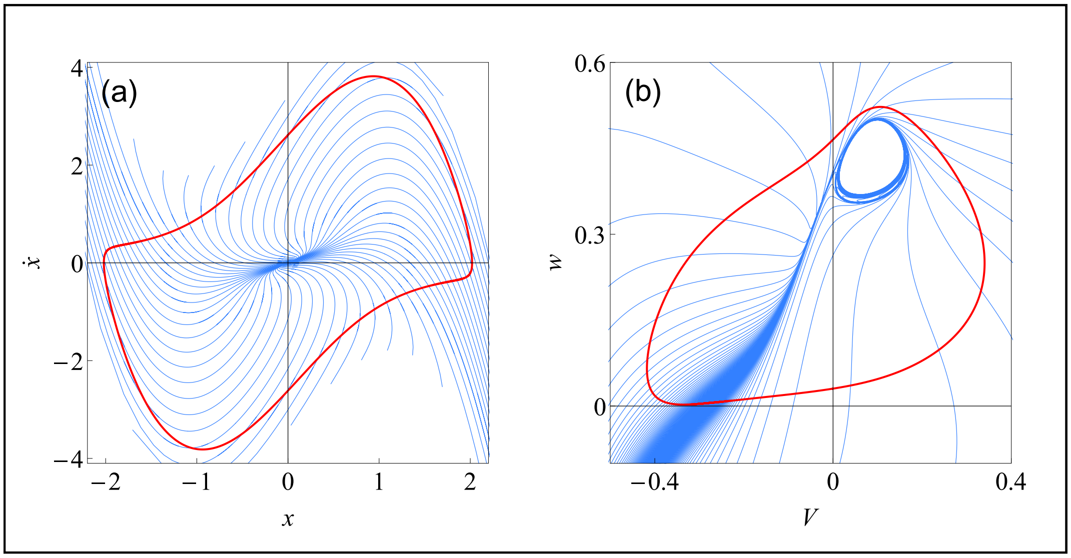

Figure S6. The isochrone structure of the two oscillators used in the main text, (a): van der Pol, Eq. (9) and (b): Morris-Lecar, Eq. (7). The limit cycle is plotted with a thick red line while the isochrones with thin blue ones. In (a) there are 50 isochrones corresponding to equal phase intervals, while in (b) there are 200.

Now suppose we only measure one variable $x(t)$ but want to embed our signal in 2 dimensions and then determine the periods using a Poincaré section at an arbitrary angle α. This is done by rotating the embedded limit cycle and calculating the threshold value accordingly, a depiction can be seen in Fig. S7.

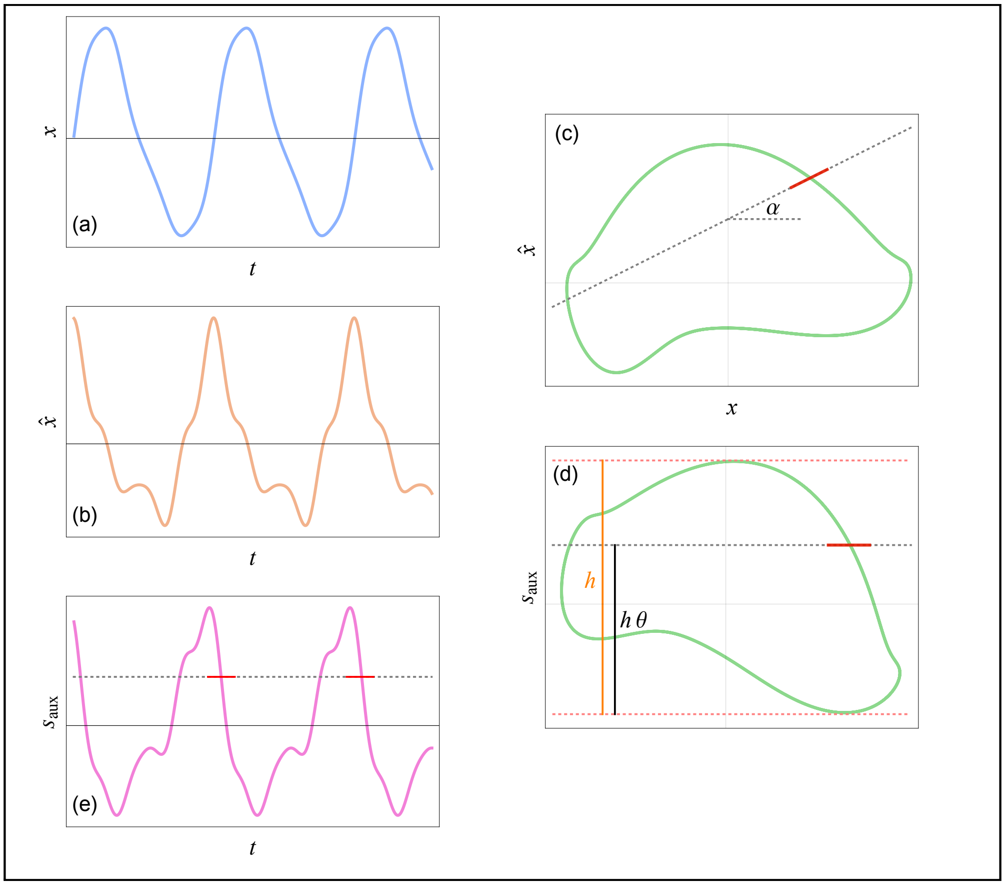

Figure S7. A schematic depiction of period determination with the use of signal embedding and inclined surfaces of section, as used for Fig. 6 in the main text. In (a) the measured signal $x(t)$ and in (b) the proxi variable $\hat{x}(t)$ (in our case we used the first derivative). In (c) the embedded signal (with green) as well as the chosen surface of section depicted with a straight red line. In (d) the embedded signal rotated by α so that the chosen surface of section becomes paralel to the horizontal axis. In (e) the vertical projection of the rotated embedded signal which is to be thresholded.

Using a similar search to that performed in the main text in Fig. 6, we can estimate the isochrone structure in the vicinity of the limit cycle, see Fig. S8. First, we embed our signl $x(t)$ in two dimensions (see Fig. S7 above). Then, considering the embedded signal in polar cooridnates, we average the radius variable over the angle variable with finite binning to obtain an approximation of the unpertubed limit cycle. On the obtained limit cycle approximation we choose points uniformly distributed in angle and for each point try several Poincaré sections at different angles. The angle corresponding to the lowest error should closely match the local isochrone.

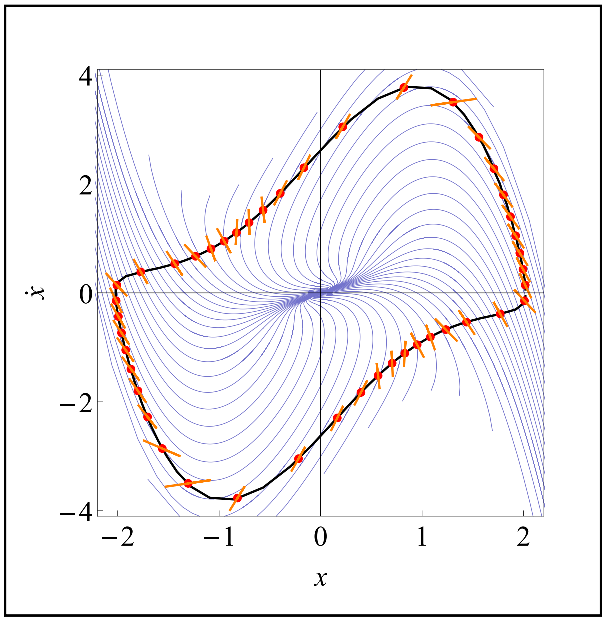

Figure S8. An estimated local isochrone structure using only one time series of length $t_{\text{sim}} = 500$ (coresponding to roughly 500 periods). The true isochrones are depicted with thin purple curves. The points where the inclination of the isochrones is estimated (in red) are uniformly distributed in angle. The estimated isochrones are depicted with orange lines. Parameters are $\varepsilon \|Z\| = 1$ and $\tau = 0.01$.

References

1. Milstein, G. N. Approximate integration of stochastic differential equations. *Teoriya Veroyatnostei i ee Primeneniya* **19**, 583–588 (1974).

2. Hindmarsh, J. L. & Rose, R. M. A model of neuronal bursting using three coupled first order differential equations. *Proceedings of the Royal Society of London B: Biological Sciences* **221**, 87–102 (1984).

3. Josic, K., Shea-Brown, E. T. & Moehlis, J. Isochron. *Scholarpedia* **1**, 1361 (2006).

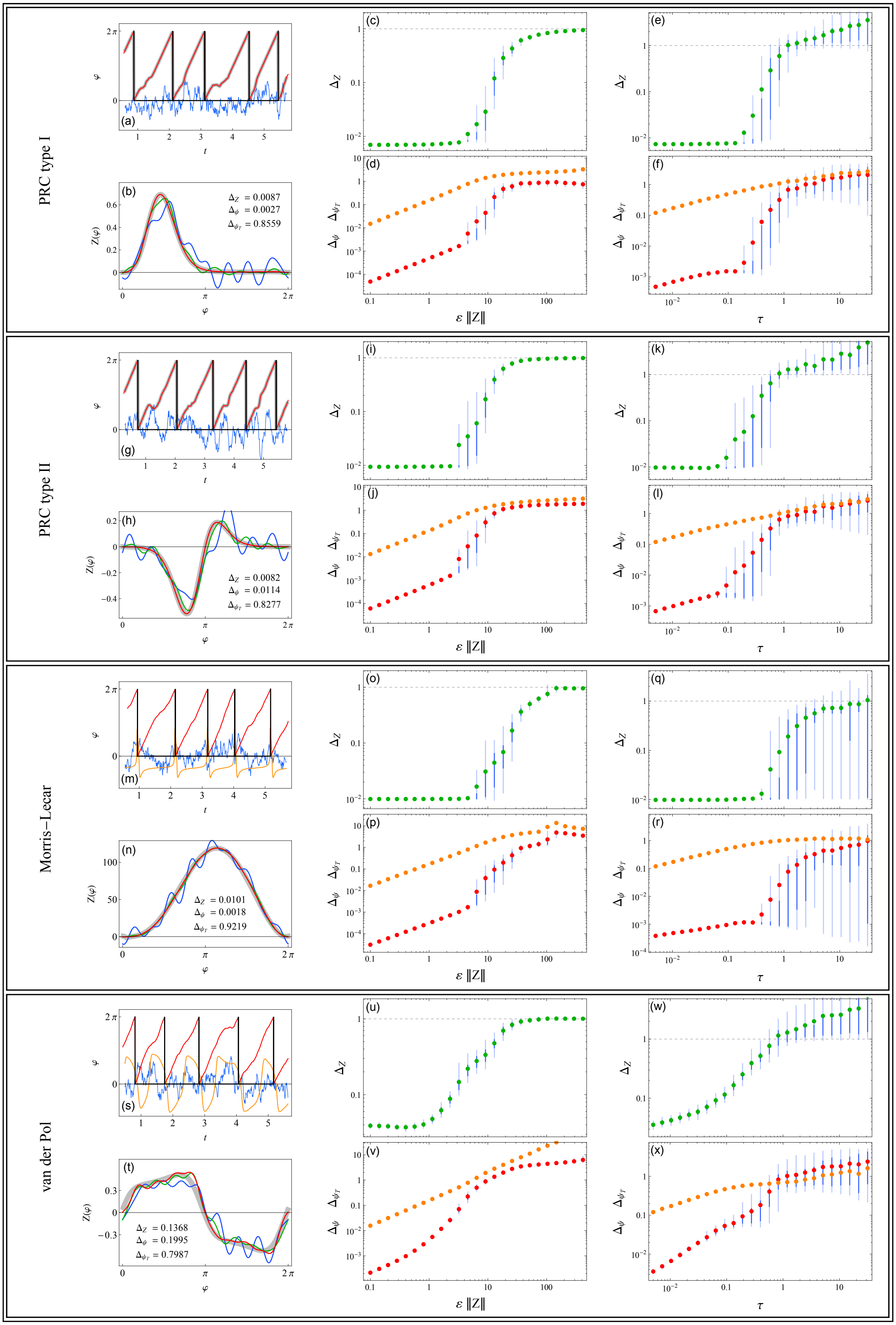

Figure S9. Overview comparison of test oscillators: PRC type I, PRC type II, Morris-Lecar and van der Pol. In plots (a, g, m, s) the signal, in (b, h, n, t) the PRC reconstruction, in (c, d, i, j, o, p, u, v) the effect of driving amplitude ε and in (e, f, k, l, q, r, w, x) the effect of driving correlation time τ. The parameters are $\varepsilon\|Z\| = 5$ and $\tau = 0.1$ in (a, b, g, h, m, n, s, t), $\tau = 0.1$ in (c, d, i, j, o, p, u, v) and $\varepsilon\|Z\| = 3$ in (e, f, k, l, q, r, w, x).
